# The Digital Transformation of Healthcare Through Intelligent Technologies: A Path Dependence-Augmented–Unified Theory of Acceptance and Use of Technology Model for Clinical Decision Support Systems

**DOI:** 10.3390/healthcare13111222

**Published:** 2025-05-22

**Authors:** Șerban Andrei Marinescu, Ionica Oncioiu, Adrian-Ionuț Ghibanu

**Affiliations:** 1 Oncological Institute “Alexandru Trestioreanu” Bucharest, 252 Soseaua Fundeni, 022328 Bucharest, Romania; serban.marinescu@yahoo.com; 2 Department of Informatics, Faculty of Informatics, Titu Maiorescu University, 189 Calea Vacaresti St., 040051 Bucharest, Romania; 3 Academy of Romanian Scientists, 3 Ilfov, 050044 Bucharest, Romania; 4 Faculty of Economic Sciences, Valahia University of Targoviste, 2 Carol I Blvd., 130024 Targoviste, Romania; aighibanu@gmail.com

**Keywords:** clinical decision support systems, digital transformation in medicine, technology acceptance in healthcare, healthcare professional attitudes

## Abstract

**Background/Objectives:** Integrating Artificial Intelligence Clinical Decision Support Systems (AI-CDSSs) into healthcare can improve diagnostic accuracy, optimize clinical workflows, and support evidence-based medical decision-making. However, the adoption of AI-CDSSs remains uneven, influenced by technological, organizational, and perceptual factors. This study, conducted between November 2024 and February 2025, analyzes the determinants of AI-CDSS adoption among healthcare professionals through investigating the impacts of perceived benefits, technological costs, and social and institutional influence, as well as the transparency and control of algorithms, using an adapted Path Dependence-Augmented–Unified Theory of Acceptance and Use of Technology model. **Methods**: This research was conducted through a cross-sectional study, employing a structured questionnaire administered to a sample of 440 healthcare professionals selected using a stratified sampling methodology. Data were collected via specialized platforms and analyzed using structural equation modeling (PLS-SEM) to examine the relationships between variables and the impacts of key factors on the intention to adopt AI-CDSSs. **Results**: The findings highlight that the perceived benefits of AI-CDSSs are the strongest predictor of intention to adopt AI-CDSSs, while technology effort cost negatively impacts attitudes toward AI-CDSSs. Additionally, social and institutional influence fosters acceptance, whereas perceived control and transparency over AI enhance trust, reinforcing the necessity for explainable and clinician-supervised AI systems. **Conclusions**: This study confirms that the intention to adopt AI-CDSSs in healthcare depends on the perception of utility, technological accessibility, and system transparency. The creation of interpretable and adaptive AI architectures, along with training programs dedicated to healthcare professionals, represents measures enhancing the degree of acceptance.

## 1. Introduction

Technological advancements and the digitalization of healthcare systems have led to the rapid expansion of artificial intelligence (AI), fostering the development of innovative solutions to optimize clinical decision-making processes [1,2,3]. In this context, Artificial Intelligence Clinical Decision Support Systems (AI-CDSSs) have emerged as promising tools for enhancing diagnostic accuracy, streamlining clinical workflows, and supporting evidence-based therapeutic decisions [4,5,6]. Even so, despite their theoretical benefits, the implementation of AI-CDSSs in medical practice remains a complex process, influenced by multiple technological, organizational, and perceptual variables [7,8,9]. This reality highlights the need for a thorough analysis of the factors shaping AI-CDSS adoption, ensuring that their integration is not only feasible but also sustainable in the long term [10,11].

A key point of discussion in the scientific literature on AI-CDSS adoption is the balance between algorithmic autonomy and the need for human assistance in medical decision-making [12,13,14,15]. While some studies contend that artificial intelligence may improve diagnostic accuracy and the efficiency of treatment beyond human knowledge in some situations, others caution against the dangers of too much dependence on automated systems, stressing the need for ongoing professional supervision [16,17,18]. In this respect, differing views remain regarding the degree to which artificial intelligence should be seen as a rigorous support system for medical practitioners or an autonomous decision-making tool.

Previous studies have shown that implementing AI-CDSSs is directly affected by healthcare professionals’ views of them [19,20,21,22]. Key drivers of adoption include the perceived advantages of artificial intelligence, such as lowering medical mistakes and streamlining decision-making [23,24,25]. However, extensive AI-CDSS deployment may be hampered by technological expenses, integration challenges with existing digital infrastructure, and the need for more knowledge [26,27,28]. Social and institutional influence—including management support and clinical guideline recommendations—also helps to promote the adoption of these systems in medical settings.

Although there is increasing scholarly interest in the use of artificial intelligence in medicine, controversy still exists regarding the main elements driving the adoption of this technology [29,30,31]. Supporters of AI underline its ability to improve the accuracy of medical decisions, lower the cognitive strain on healthcare professionals, and cut down on diagnostic mistakes. Institutional backing and societal pressure may also either speed up or slow down the use of artificial intelligence systems in clinical practice.

Building on these considerations, the present study aims to address the following research questions:

RQ_1_: How do the perceived costs and benefits of AI-CDSSs influence healthcare professionals’ attitudes and intentions to adopt these systems?

RQ_2_: To what extent do social influence and institutional support drive AI-CDSS adoption?

RQ3: What is the relationship between attitudes toward AI-CDSSs, perceived control, and the intention to use AI?

RQ4: How do transparency and control over AI affect the perception of benefits and the use of AI-CDSS?

This research explores the relationship between perceived control over artificial intelligence, general professional attitude, and intention to use AI-CDSSs in medical practice. The originality of this study lies in its multidimensional approach to understanding the adoption of AI-CDSSs among healthcare professionals. Grounded in an adapted Path Dependence-Augmented–Unified Theory of Acceptance and Use of Technology (PDA-UTAUT) framework, the research moves beyond prior studies that have mainly focused on either technological advantages or structural barriers. Instead, it captures the interplay between subjective perceptions—such as decision-making control, algorithmic transparency, and perceived effort—and broader institutional and social dynamics that shape acceptance in clinical settings. By positioning these factors within a unified theoretical model, the study provides a more comprehensive view of the mechanisms underlying AI-CDSS adoption in modern medical practice.

## 2. Literature Review and Hypothesis Development

### 2.1. AI-CDSSs and the Digitalization of Clinical Decision-Making in Healthcare

The digital transformation of healthcare systems has changed clinical decision-making by offering creative ways to improve medical procedures [32,33,34]. Integrating Artificial Intelligence Clinical Decision Support Systems (AI-CDSSs) into healthcare is a major development, as it helps to optimize clinical operations, increase diagnostic accuracy, and tailor therapies [35,36]. These systems immediately affect the quality of patient treatment by helping healthcare professionals understand clinical data, interpret them, and optimize resources within medical institutions [37].

Rule-based systems meant to assist clinical judgments via predefined algorithms started the evolution of AI-CDSSs. However, advancements in machine learning technology have led to the creation of systems that can analyze vast amounts of medical data, identify relevant patterns, and offer tailored recommendations [38]. Hospitals, for example, have used artificial intelligence to forecast patient problems, optimize treatment plans, and halt decline. The treatment of chronic patients has been greatly enhanced by the integration of artificial intelligence into digital health systems, which has also lowered the time needed for key decision-making in intensive care units.

AI-CDSSs have important uses in healthcare for medical picture analysis in radiology, dermatology, and ophthalmology. Contributing to the early detection of cancer, cardiovascular illnesses, and retinal problems, AI systems can identify pathological abnormalities with an accuracy equivalent to that of medical professionals [39,40,41]. These developments facilitate the integration of AI into medical processes, thereby raising questions about the optimal level of human involvement in AI-assisted decision-making.

In the healthcare setting, artificial intelligence-powered monitoring systems allow for the early detection of indicators of patient decline, proactively avoiding problems [42,43,44]. Artificial intelligence in electronic health records also helps to automatically examine patient medical histories, supporting individualized therapy and lowering negative treatment responses. Building user trust and guaranteeing the efficient integration of artificial intelligence into healthcare systems depend on the creation of explainable artificial intelligence systems with unambiguous justifications for every proposed medical action [45]. AI integration in healthcare will thus be successful depending on the capacity of health systems to fit AI-CDSSs to actual clinical demands, thus encouraging the responsible and efficient use of artificial intelligence in medicine.

### 2.2. Theoretical Frameworks

Adopting AI-CDSSs in medicine is a difficult process that is influenced by many technological, organizational, and perceptual elements. To better comprehend this dynamic, some authors have relied on different theoretical frameworks developed to elucidate the acceptance and use of emerging technologies [46,47,48]. Among the most pertinent models in this regard are the Technology Acceptance Model (TAM), the Unified Theory of Acceptance and Use of Technology (UTAUT), and the Information Systems Success Model (IS Success Model). These conceptual frameworks enable one to see the factors influencing the attitudes and behaviors of healthcare professionals toward AI-CDSSs, supporting the creation of efficient plans for including these systems in clinical practice [49].

Proposed by Davis (1989) [50], the TAM stresses how perceived utility and simplicity of use shape the intention to embrace a technology. Medical professionals are more likely to employ AI-CDSSs if they believe they increase diagnostic accuracy and lower medical mistakes [51,52]. However, their usage can be postponed or even rejected if these technologies are considered challenging to use or correspond to current processes [49,53].

From a wider angle, the UTAUT, created by Venkatesh et al. (2003) [54], presents four key elements affecting the adoption of digital technologies: performance expectations, effort expectations, social influence, and facilitating conditions. This theory implies that healthcare professionals are more likely to embrace AI-CDSSs if they see them as efficient and user-friendly, receive support from colleagues and medical guidelines, and have access to the required infrastructure for implementation [52,55,56].

The IS Success Model is another important theoretical framework that examines how information quality and system usability affect user happiness and organizational advantages [57]. Trust in AI-CDSSs is influenced by the quality of algorithms, the openness of decision-making processes, and users’ capacity to grasp the logic behind suggestions. Yusof et al. (2008) [58] indicate that the impression of control and algorithmic openness favorably affects adoption intention, stressing the need for explainable artificial intelligence systems enabling human engagement in medical decision-making.

Recently, the PDA-UTAUT model was modified to investigate how users’ earlier experiences shape their adoption and view of new technology [59]. This model offers a useful viewpoint on how healthcare professionals’ past exposure to digital technology influences their willingness to embrace AI-CDSSs. For instance, medical professionals who have utilized electronic health record systems may be more likely to embrace AI-CDSSs, as they see them as a logical extension of technology already in place.

### 2.3. Intention to Adopt AI-CDSSs

Studies on the need to use AI-CDSSs have shown various elements during the past five years that influence the integration of these technologies into medical practice [16,21]. AI-CDSS adoption is a complicated, non-linear process shaped by technical, organizational, and perceptual factors affecting healthcare professionals’ choices [60,61]. AI models used in the medical industry have to provide obvious and understandable arguments for their recommendations, thereby guaranteeing that professionals can grasp the reasoning behind the decisions and maintain control over the medical act [16].

The efficacy of these systems relies on their capacity to fit into current digital infrastructures and provide pertinent information without interfering with the physician’s decision-making processes [62,63]. Models that are successfully applied lower the cognitive burden for users and maximize decision-making by automating monotonous chores, thereby enabling fast and well-founded therapeutic treatments [21].

Davenport and Kalakota (2019) [61] emphasized that when healthcare professionals see these systems as useful support tools in clinical decision-making, they are more likely to use them, hence lowering mistakes and enhancing diagnostic accuracy. On the other hand, doubts over AI’s actual influence on operational efficiency and the quality of patient care can postpone the use of these technologies.

Thus, AI-CDSS adoption is a multidimensional process that requires solutions tailored to the real needs of healthcare professionals. Developing explainable systems that are efficiently integrated into clinical workflows and supported by institutional policies and training programs represents a strategic approach to accelerating AI adoption in medicine. Given these challenges, a balanced approach is imperative—one that maximizes the benefits of AI-CDSSs while maintaining the necessary safety and ethical standards in contemporary medical practice.

### 2.4. Attitudes Toward AI-CDSSs

From the perspective of the specialized literature, a favorable attitude toward AI-CDSSs can be defined as healthcare professionals’ positive perception and trust in these systems. This significantly influences their intention to integrate them into clinical practice [64]. Wrzosek et al. (2020) [65] demonstrated that the acceptance of artificial intelligence-based technologies is closely linked to their perceived usefulness, effectiveness in reducing medical errors, and capacity to optimize decision-making. A positive attitude toward AI-CDSSs not only fosters a stronger intention to use these systems but also facilitates a smoother transition toward their integration into existing clinical workflows.

Investigations in this field suggest that medical professionals with a positive attitude toward artificial intelligence are more likely to see these systems as complementing rather than supplanting their clinical experience [21,60]. Furthermore, the acceptability of AI-CDSSs is greatly influenced by past favorable encounters with digital technologies, helping to integrate them smoothly into medical settings [66]. Based on these previous studies, the following hypothesis is proposed:

**Hypothesis 1 (H_1_).**  *Attitudes toward AI-CDSSs positively influence the intention to adopt AI-CDSSs.*

### 2.5. Technology Effort Cost

Technological difficulty can discourage the implementation of these systems, as healthcare professionals usually struggle with new digital technologies, especially those needing sophisticated technical expertise and workflow changes [13,26,43]. The steep learning curve associated with AI-CDSSs, coupled with the potential need for continuous updates and system maintenance, can create resistance among users who perceive these technologies as difficult to integrate into their daily clinical routines.

Institutions with limited financial and technical resources may find it difficult to provide enough money for the smooth deployment of AI-driven decision support systems, hence postponing or even prohibiting their integration. Given this, healthcare professionals may not trust an AI-CDSS as much if they think it will cost a lot, both in terms of money and the mental work needed to learn how to use it. Fostering a more positive attitude and enabling broad adoption through focused training, user-friendly interfaces, and institutional support systems is necessary to address these issues [66]. Therefore, the current study proposes the following hypothesis:

**Hypothesis 2 (H_2_).**  *Technology effort cost negatively influences attitudes toward AI-CDSSs.*

Apart from technical difficulties, the financial pressure linked to AI-CDSSs’ deployment might impede their widespread use. Significant challenges include the high expenditures of the required IT infrastructure, software, and ongoing staff training [26]. The use of AI-CDSSs is limited in clinics, even when their benefits are clear, due to the need for institutional support policies and long-term financing models [27].

Moreover, the perception that AI may increase the administrative workload and require significant workflow adaptations contributes to greater resistance among users [44]. Therefore, to foster AI-CDSS adoption, it is important to develop strategies that address technological optimization cost reduction, enhanced accessibility, and the provision of tailored training programs that align with the specific needs of healthcare professionals. The following is the third hypothesis of this study:

**Hypothesis 3 (H_3_).**  *Technology effort cost negatively influences the intention to adopt AI-CDSSs.*

### 2.6. Perceived Benefits of AI-CDSSs

Reducing errors in medicine using AI-CDSSs also increases user confidence in these systems, strengthening the readiness for using them [67]. By offering suggestions that depend on the most recent scientific information, AI-CDSSs facilitate more informed therapeutic decision-making, thus aiding in the standardization of medical procedures and lowering treatment variability [68]. Attitudes toward bringing artificial intelligence into clinical practice become more positive as more healthcare providers see these benefits.

The authors suggest that incorrect clinical judgments account for a notable percentage of patient care mistakes, either from the sheer amount of information medical professionals must handle or the natural cognitive constraints of human decision-making [36]. AI-CDSSs serve as decision support tools, providing clinicians with alerts and suggestions based on machine learning algorithms, thus helping to prevent incorrect prescriptions or critical diagnostic omissions. Thus, this study proposes the following hypothesis:

**Hypothesis 4 (H_4_).**  *The perceived benefits of AI-CDSSs positively influence attitudes toward AI-CDSSs.*

Despite this, the perception of AI-CDSS complexity and the resources required for implementation may temper initial enthusiasm and lead to a more skeptical assessment of their benefits. Healthcare professionals may view these systems as difficult to use or requiring an extended training period, which can generate reluctance toward adoption [65]. Including AI-CDSSs in the current system of hospitals and clinics could be rather expensive and necessitate changes in processes, thus becoming another obstacle to technology acceptability [68]. Should these issues go unaddressed, consumers could assess the value of AI-CDSSs more warily, as the benefits provided do not offset the required implementation effort and expenditure. In addition, a high level of perceived technological effort is frequently associated with a reduction in perceived benefits, as users tend to focus more on the immediate difficulties than on the potential advantages of the technology. This assumed negative relationship between the cost of technological effort and perceived benefits still requires solid empirical justification, as existing data are limited or indirect [26]. Hence, the hypotheses are as follows:

**Hypothesis 5 (H_5_).**  *The perceived benefits of AI-CDSSs positively influence the intention to adopt AI-CDSSs.*

**Hypothesis 6 (H_6_).**  *Technology effort cost negatively influences the perceived benefits of AI-CDSSs.*

### 2.7. Social and Institutional Influence

Medical institutions that promote the integration of emerging technologies through clear policies, investments in infrastructure, and professional training programs create a favorable environment for the use of these systems [69]. AI-CDSS acceptance is supported not only by the perceived benefits of this technology, but also by the confidence that it is validated and endorsed by healthcare leadership structures. Studies indicate that hospitals and clinics that implement standardized protocols for the use of AI in clinical decision-making experience a higher degree of acceptance of new systems among medical staff [2,66].

Endorsements from medical associations, specialty societies, and medical ethics committees have a considerable influence on practitioners’ clinical decisions. In this regard, including AI-CDSSs in international medical practice standards and recognizing their effectiveness in clinical guidelines contribute to reducing resistance to change and to its acceptance as a complementary tool to human expertise [45]. Given this, institutional support and professional validation facilitate the transition to the use of AI in healthcare, enhancing trust in these systems and reinforcing their sustainable integration into clinical practice. As a result, the following hypothesis is proposed:

**Hypothesis 7 (H_7_).**  *Social and institutional influence positively impacts attitudes toward AI-CDSSs.*

Furthermore, the endorsement of AI-CDSSs by healthcare institutions and their integration into organizational policies significantly contribute to the large-scale adoption of this technology. Hospitals and clinics that implement clear digitalization strategies and allocate resources for staff training in AI-CDSS usage create a favorable environment for the acceptance of these systems [70]. Therefore, the active support of key opinion leaders and healthcare institutions not only facilitates the integration of AI-CDSSs into clinical practice but also accelerates the digital transformation of the medical system, ensuring the efficient and sustainable implementation of new technologies for the benefit of both patients and healthcare professionals. Thus, the proposed hypothesis is as follows:

**Hypothesis 8 (H_8_).**  *Social and institutional influence positively impacts the intention to adopt AI-CDSSs.*

### 2.8. Perceived Control and Transparency over AI

Transparency, defined as a system’s ability to provide clear and comprehensible explanations of its decision-making rationale, is a key factor in strengthening user trust [33,71]. In a clinical context, the lack of clarity regarding how AI-CDSSs generate recommendations can lead to skepticism and reluctance to use, thereby affecting the system’s acceptance rate [72]. Conversely, algorithm explainability allows users to assess the validity of the proposed decisions, reducing perceived technological risk and enhancing the sense of professional security [67].

Systems that offer transparent justifications for decision-making and ensure interoperability with human medical expertise are more likely to be adopted and effectively integrated into existing clinical workflows. Consequently, the development of AI-CDSSs should be based on a technological architecture that prioritizes both explainability and the capacity for control and adjustment, ensuring that these systems function as complementary tools in clinical decision-making, striking an optimal balance between automation and human expertise. Given this, the proposed hypothesis is as follows:

**Hypothesis 9 (H_9_).**  *Perceived control and transparency over AI positively influence attitudes toward AI-CDSSs.*

Systems with a high degree of explainability reduce perceived uncertainty, facilitating a more efficient integration of artificial intelligence into medical decision-making and alleviating hesitations associated with the use of algorithmic technologies in healthcare [69]. Without obvious predictability, artificial intelligence may be seen as an unstable system, undermining the confidence of healthcare professionals and postponing its adoption into clinical procedures [73]. However, when AI-CDSSs operate on clear and repeatable algorithms, medical professionals are more likely to use these systems, as they see them as efficient and feasible decision support tools [14].

Thus, the evolution of AI-CDSSs ought to prioritize explainability and predictability, thereby guaranteeing a seamless transition toward the broad acceptance of these systems in medical practice. Improving the quality of clinical decision-making and strengthening patient safety are the immediate advantages of this strategy. Thus, the next hypothesis of this study is as follows:

**Hypothesis 10 (H_10_).**  *Perceived control and transparency over AI positively influence the intention to adopt AI-CDSSs.*

## 3. Research Methodology

Focusing on five important aspects—the perceived benefits of AI-CDSSs (PBAI), the technological effort cost required for implementation (TEC), attitude toward AI-CDSSs (ATAI), social and institutional influence (INF), and the perceived control and transparency over AI (CTRL)—this study investigates the multifaceted elements influencing the adoption of AI-CDSSs among healthcare professionals. This research aims to provide nuanced knowledge of what motivates—or impedes—the desire of medical professionals to include AI-CDSSs in their clinical workflows through an analysis of these related factors.

Furthermore, this study was conducted utilizing a cross-sectional approach and a stratified sampling technique to select a sample of 440 healthcare professionals, who answered these questions through a structured questionnaire. Partial Least Squares Structural Equation Modeling (PLS-SEM) was used to analyze the gathered data, enabling an evaluation of correlations between variables and the identification of the main drivers of AI-CDSS adoption.

Using a modified PDA-UTAUT model, this study further helps to clarify the processes supporting the adoption of AI-CDSSs by healthcare practitioners. Unlike other studies, this one provides a comprehensive view of how perceived advantages, technology expenses, social and institutional impacts, and algorithm transparency and control determine the desire to implement AI-CDSSs [32,33,34].

**Figure 1:**
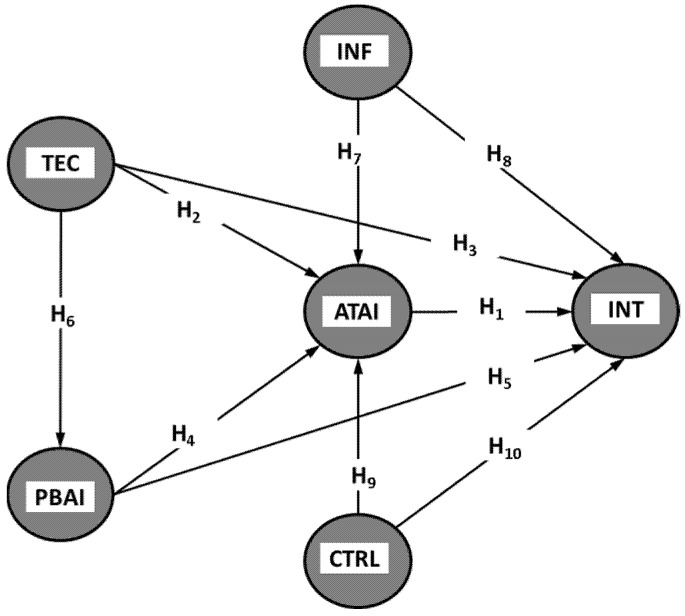
Proposed research model.

The suggested study model, represented in Figure 1, illustrates the dynamic interaction between these drivers, enabling a holistic view of how AI-CDSS adoption is influenced by real-world healthcare environments.

This study used an organized method to develop survey questions based on known theoretical frameworks and insights from prior empirical investigations to guarantee scientific rigor and validity. These issues have been contextualized and honed to fit the particular realities of AI-CDSSs in the healthcare field. Per Table 1, the scales used to measure core concepts have been changed from basic models in system effectiveness and technology acceptance to include viewpoints from well-known academic research [50,54,57].

This approach draws on a thorough examination of the literature, enabling the inclusion of verified notions and their modifications to the changing landscape of AI adoption in clinical practice. Professional attitudes toward perceived advantages, technical effort, institutional support, and receptivity to new digital technologies have long been identified in studies [22,74]. This study provides a structured but adaptable way to think about how AI-CDSSs could be effectively added to clinical workflows by combining the difficulty of making decisions in modern healthcare with tried-and-true theoretical knowledge.

The data collection process took place between November 2024 and February 2025, with participants selected through a random stratified sampling methodology using the UpToDate [75] and Data Sweep [76] platforms, professional resources dedicated to medical professionals and healthcare experts. These platforms provide access to the latest scientific evidence and support clinical decision-making, ensuring a relevant selection of respondents while minimizing potential biases associated with conventional sampling methods.

The questionnaire was conducted as an anonymous online survey distributed to healthcare professionals who could directly or indirectly come into contact with AI-CDSSs. Participants are exclusively from Romania and work in public and private healthcare settings, covering a variety of clinical specialties. This institutional and professional distribution was essential to capture diverse perspectives on the use of AI-CDSSs in real-world healthcare settings, reflecting both public and private sector experiences. Selecting respondents from both settings contributed to a more nuanced understanding of the factors influencing acceptance and adoption intentions for these technologies. Perceptions and adoption intentions were measured using a five-point Likert scale; 1 was “strongly disagree”, and 5 was “strongly agree”.

The first questionnaire went through a pre-testing stage, with 20 digital health experts chosen for their knowledge of using new technologies and digitizing medical services to guarantee the accuracy, consistency, and usefulness of the study tool. This stage precisely calibrated the questions to properly reflect the actual difficulties related to AI-CDSS adoption in many healthcare industries. Moreover, the changes ensured that the gathered answers provided relevant and significant insights, thereby supporting better knowledge of the elements affecting AI-CDSS integration in clinical workflows and healthcare service delivery.

Survey distribution was accomplished using the snowball sampling technique, which guaranteed a varied response pool and fair distribution of respondents while also enabling natural participation growth. Subsequent statistical analysis was supported by this strong empirical database, which also tests the PDA-UTAUT model used to find the main drivers of AI-CDSS adoption.

This investigation covered various fields where artificial intelligence improves medical decision-making to provide a more comprehensive view of the relevance of AI-CDSSs in healthcare services. These services included pulmonology, gastrointestinal care, anesthesiology and critical care, robotic and minimally invasive surgery, endocrinology, and neurology. The inclusion of artificial intelligence in various fields shows the variety of clinical issues and the intricacy of medical decision-making, enabling the best treatment approaches and the individualization of patient care.

The growing use of AI-CDSSs in many medical sectors highlights their importance in decreasing medical mistakes, increasing diagnostic efficiency, and raising the standard of medical treatment. Implementing these technologies helps the healthcare system to adapt to digital changes and provides healthcare professionals with sophisticated tools for making well-informed, evidence-based options.

SmartPLS 4—noted for its adaptability in handling non-normally distributed datasets and its efficiency in studies with modest sample sizes—was used for data analysis and to validate both the measurement and structural models. As it is commonly used in studies on the adoption of emerging healthcare technologies, the PLS-SEM approach was selected for its capacity to handle complicated models with several relationships between latent variables and its efficiency in maximizing estimation accuracy under non-normal data distribution conditions.

While convergent validity was evaluated via factor loadings and the average variance extracted (AVE), reliability was measured using Cronbach’s α coefficient and composite reliability (CR). Equally, discriminant validity was confirmed using the Fornell–Larcker criterion and the Heterotrait–Monotrait (HTMT) ratio, guaranteeing the conceptual separation of the AI-CDSS model components.

## 4. Results

Of an original sample of 540 people, 440 participants effectively finished the survey; thus, following this procedure, it produced a response rate of 81.5%. The sample distribution and demographic characteristics of the respondents are presented in Table 2, illustrating the representativeness and justification of the analyzed professional categories.

The healthcare professional categories include specialist physicians, resident doctors, and nurses who directly use AI-CDSSs. The chosen specialties reflect areas where artificial intelligence greatly affects the medical decision-making process, facilitating diagnosis, treatment planning, and intervention optimization. These include radiology, cardiology, oncology, and emergency care, where artificial intelligence helps experts analyze medical pictures, spot risk indicators, and improve diagnostic accuracy. Healthcare professionals in these areas help to integrate artificial intelligence into clinical practice by having a direct and practical view of the advantages and limits of this technology.

The results are summarized in Table 3, which presents the reliability and validity indicators associated with each construct analyzed in this study. The values of Cronbach’s α and CR exceed the threshold of 0.7 for all examined constructs, indicating an adequate level of internal consistency within the scale. This outcome confirms that the items associated with each construct measure the same conceptual dimensions. In addition, the AVE values are above the 0.5 threshold, demonstrating that each construct adequately captures the variance of its indicators. Additionally, most items exhibit factor loadings above 0.75, reinforcing the convergent validity of the model.

To validate the measurement model used in this study, three well-established methods were applied to assess discriminant validity: the Fornell–Larcker criterion, indicator cross-loadings, and the Heterotrait–Monotrait criterion. These techniques ensure that each construct is conceptually and statistically distinct from the other variables included in the model, thereby reinforcing the methodological robustness of the analysis concerning AI-CDSS adoption by healthcare professionals.

In the first stage, the Fornell–Larcker criterion was employed to verify whether the square root of the AVE for each construct is greater than its correlations with other model variables. The results presented in Table 4 confirm this criterion for all analyzed variables, indicating that each construct is well defined conceptually. For instance, INT (0.88) has a higher value than any of its correlations with other variables, confirming that the intention to adopt AI-CDSSs is distinct from the other dimensions of the model. Furthermore, the moderate correlations between variables suggest an interdependence among factors without conceptual overlaps, supporting the robustness of the measurement model.

In the second stage, the cross-loadings of the indicators were used to validate the discriminant validity of the measurement model. According to the applied methodology, discriminant validity is confirmed if each indicator has a higher factor loading on its respective construct than on any other construct in the model.

In Table 5, the factor loadings on their respective variables are high, confirming that each item significantly contributes to defining its construct. At the same time, the cross-loadings on other constructs are significantly lower, indicating strong discriminant validity. Consequently, TEC exhibits negative correlations with INT and ATAI, supporting the hypothesis that a high technological effort cost reduces both the intention to adopt and a favorable attitude toward AI-CDSSs. Conversely, INF and CTRL are closely related to INT, therefore confirming that social support and the perception of control and algorithm openness affect the adoption of AI-CDSSs positively. These results support the use of this model to investigate the relationships between variables free from the risk of conceptual overlap across dimensions.

In the final stage, the HTMT criterion was employed for an additional assessment of discriminant validity. This criterion requires corresponding HTMT coefficients against a specified threshold; results beyond this level suggest a possible lack of discriminant validity. Current studies point to two traditional thresholds: 0.85 for models with closely linked components and 0.90 for wider models with more independent variables. The findings reveal that all HTMT values, except for one, are below the 0.85 threshold, and all values stay under 0.90, hence verifying the discriminant validity of the measurement model. The reflective structures’ HTMT ratios are shown in Table 6. The highest HTMT coefficient values can be seen between PBAI and ATAI (0.62) and between INF and INT (0.59), suggesting a greater link between these variables without affecting discriminant validity.

**Figure 2:**
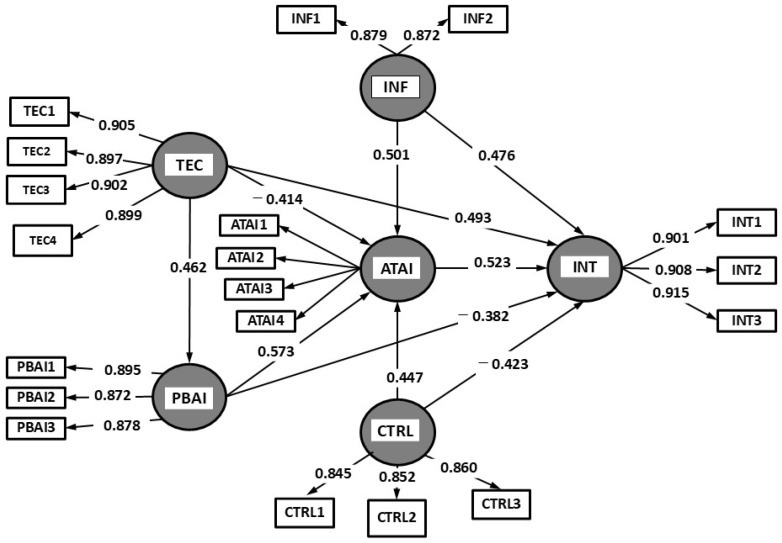
Results of path analysis.

The hypothesis testing of the PDA-UTAUT model confirms that all relationships between variables are statistically significant (*p* < 0.001), with path coefficients (*β*) indicating the strength of these associations. Conversely, negative relationships suggest that the perception of technological costs may diminish the perceived benefits of AI, potentially discouraging its use. The effect size (*f*^2^) further confirms that the significant relationships exert a moderate to strong impact on the dependent variable (INT), reinforcing the validity of the model. Correspondingly, the R^2^ value and confidence intervals are employed to validate the structural paths of the conceptual model. The results in Figure 2 assume that all hypotheses are supported with a significance of *p* = 0.05.

The analysis of the AI-CDSS model confirms the validity of the proposed hypotheses, demonstrating significant relationships between the studied variables. The perceived benefits of AI-CDSSs exert a strong positive influence on adoption intention (H_1_: *β* = 0.523, *p* < 0.001), supporting the hypothesis that perceived utility drives users’ predisposition to integrate these systems into medical practice. This finding suggests that healthcare professionals are more inclined to adopt AI-CDSSs when they recognize their potential to enhance clinical decision-making. The relevance of this relationship underscores the necessity of promoting AI-CDSS advantages, such as reducing medical errors, optimizing diagnostic time, and supporting complex clinical decisions.

Similarly, perceived technological costs negatively impact attitudes toward AI-CDSSs (H_2_: *β* = −0.414, *p* < 0.001), confirming that technological barriers can discourage adoption. Furthermore, user attitude has proven to be a strong predictor of AI-CDSS usage intention (H_3_: *β* = 0.493, *p* < 0.001), indicating that a favorable perception of the technology is a key determinant in the acceptance process.

Perceived benefits directly and positively influence users’ attitudes toward AI-CDSSs (H_4_: *β* = 0.573, *p* < 0.001), whereas perceived technological costs negatively affect both attitudes toward AI and perceptions of AI-CDSS benefits (H_5_: *β* = −0.382, *p* < 0.001). Consequently, perceived cost acts as a deterrent, emphasizing the need for more efficient and accessible integration strategies for healthcare professionals. Additionally, user attitude significantly contributes to the perception of AI control and transparency (H_6_: *β* = 0.462, *p* < 0.001), indicating that individuals with a positive opinion of AI-CDSSs perceive these systems as more predictable and controllable.

Social and institutional influence emerged as a key factor in shaping both attitudes (H_7_: *β* = 0.501, *p* < 0.001) and adoption intention (H_8_: *β* = 0.476, *p* < 0.001), highlighting the role of professional communities and regulatory frameworks in facilitating the use of these technologies. Equally important, perceived control over AI positively influences both adoption intention (H_9_: *β* = 0.447, *p* < 0.001) and attitudes toward AI-CDSSs (H_10_: *β* = 0.423, *p* < 0.001), reinforcing the importance of transparency and the capacity for human intervention in AI-driven systems.

These findings indicate that all indirect effects are significant, partially or fully confirming the mediating role of attitudes toward AI-CDSSs, as follows:

- Healthcare professionals who acknowledge the advantages of AI-CDSSs in diagnosis and treatment exhibit a more positive attitude toward these systems, increasing their adoption intention;
- AI-CDSS acceptance within medical organizations and its promotion by opinion leaders contribute to forming a favorable perception, thereby facilitating technology adoption;
- Physicians who perceive AI-CDSSs as transparent and adaptable systems are likelier to develop a positive attitude and integrate them into clinical practice.

Likewise, the findings corroborate all hypothesized correlations, indicating that the modified PDA-UTAUT model is appropriate for clarifying the elements affecting AI-CDSS adoption in the medical domain. The study thus underscores that perceptions of algorithmic transparency, societal and institutional norms, and the balance between anticipated advantages and perceived costs all define a multifaceted process of AI-CDSS adoption.

## 5. Discussion

AI-CDSS adoption was examined in the current study using a mix of strong theoretical frameworks. We offer a thorough examination of how perceived advantages, technical costs, social and institutional impacts, and the degree of control and openness of artificial intelligence systems determine AI-CDSS adoption in healthcare by combining the TAM, UTAUT, IS Success Model, and PDA-UTAUT. These points of view help to create efficient plans that enable AI integration into healthcare systems, maximizing advantages for patients and professionals.

The purpose of our research questions is to determine how the PDA-UTAUT model variables interact with each other. This will help us to fully understand the factors that influence healthcare professionals’ decisions to use AI-CDSSs. Considering both individual user perspectives and the institutional and societal factors that affect choices about AI usage, these questions are designed to cover aspects of technology integration in medical practice.

The first research question investigates how the perception of technology costs and the perceived advantages of AI-CDSSs affect the intentions of healthcare professionals to use these systems, as well as their attitudes. The perceived cost of AI-CDSSs goes beyond financial considerations to include the time needed for education, issues with workflow integration, and extra resources needed for deployment. These elements could bring about user resistance, resulting in a more unfavorable attitude toward AI-CDSSs and affecting adoption intention (H_2_, H_5_). However, the benefits that people think AI-CDSSs have, like faster decision-making, fewer medical mistakes, and more accurate diagnoses, can balance out how much people think they cost. This makes it easier for medical professionals to accept and use AI-CDSSs, especially when they need to make quick, data-driven decisions (H_1_).

Our research indicates that the perceived advantages of AI-CDSSs have a significant positive impact on adoption intentions among healthcare professionals. Users who see obvious improvements in operating efficiency and diagnostic accuracy are likelier to adopt this technology. However, the perception of technical expenses, especially those related to integrating AI-CDSSs into clinical processes, often discourages their usage. Studies indicate that attitudes and adoption intention regarding digital advances in the medical sector are mostly influenced by perceptions of usefulness and technical accessibility [36,42,45]. Some studies indicate that encouraging AI-CDSS adoption calls for policies meant to reduce technical obstacles via customized training courses and the seamless integration of these systems into the digital ecosystem of healthcare organizations [43,49].

The second research question investigates how societal and institutional pressure shapes AI-CDSS adoption. The acceptance of new technology inside a medical system relies not only on the individual perspectives of healthcare professionals but also on the organizational environment in which they operate. AI-CDSS implementation is supported by hospital leadership, worldwide clinical standards, professional groups, and peer recommendations. Policies encouraging artificial intelligence technology and its integration into standardized protocols may help speed up its adoption and use in medical practice. Policies supporting AI technologies further serve to encourage this acceptability.

While medical professionals whose colleagues were already using AI-CDSSs were more likely to embrace the technology, those who had institutional support for AI deployment disclosed a greater degree of acceptability. Our study emphasizes the need for proactive institutional policies in fostering artificial intelligence and offers the idea that mentoring programs and favorable peer experiences could accelerate the adoption of new technologies in the healthcare sector. These findings are consistent with other studies stressing the important role of social influence and organizational support in the acceptance of creative healthcare technology [52].

The third research question examines the correlation between healthcare workers’ desire to employ AI-CDSSs, perceived control over these systems, and attitudes toward them. Technology acceptance is based on the degree to which users see AI-CDSSs as reliable instruments and their capacity to maintain control over choices produced by artificial intelligence. Medical professionals are more likely to embrace artificial intelligence if they believe they can supervise, confirm, and modify AI suggestions depending on their clinical knowledge (H_3_). Beyond that, the impression of control over technology and the openness of algorithmic conclusions are two elements that might help to lower resistance to artificial intelligence in diagnosis and treatment (H_6_, H_9_).

This research demonstrates that the motivation to use AI-CDSSs is strongly influenced by a positive mindset toward artificial intelligence; this correlation is mediated by perceived control. Those who viewed AI-CDSSs as flexible tools that allow them to change algorithmic judgments were more inclined to use them. These results align with other studies emphasizing the importance of perceived control and transparency in AI acceptance in healthcare [2,56].

The last research question looks at how opinions on AI transparency and control affect the perceived advantages of an AI-CDSS and its real use in medical practice. Users have to grasp the processes by which artificial intelligence makes certain judgments about whether AI-based solutions are to be properly included in healthcare systems. While a high degree of decision-making clarity might enhance confidence in these systems, a lack of openness can raise questions about AI dependability (H_4_, H_10_).

To strengthen the interpretation of the results obtained, it is essential to highlight the explanatory power of the structural model used. The coefficient of determination (R^2^) for the ITL construct has a value of 0.60, indicating a high level of explanatory power, according to the methodological standards applicable to PLS-SEM analysis. This value reflects the fact that the predictive variables included in the model contribute significantly to explaining the decision-making behavior of healthcare professionals in relation to the adoption of AI-CDSSs, while supporting the theoretical coherence and empirical validity of the proposed conceptual framework.

In like manner, the Q^2^ value obtained for the same construct exceeds the methodological reference threshold (>0.35), confirming the predictive power of the model. This analytical performance shows that the model is not limited to a theoretical description of the relationships but reliably anticipates adoption behaviors in real clinical contexts. Therefore, the results support not only the conceptual relevance of the proposed framework but also its practical applicability in shaping targeted interventions—from professional training initiatives focused on reducing perceived technological effort to institutional strategies aimed at strengthening trust and organizational support in the process of integrating artificial intelligence into current medical practice.

From a theoretical perspective, the study highlights that although perceived benefits of AI contribute to the formation of positive attitudes, they do not automatically determine the intention to adopt. This finding highlights the importance of intermediary mechanisms, such as the existence of a favorable organizational climate and trust in AI-assisted decisions, in the adoption process. It also emphasizes the role of collaborative networks among users, which facilitate the exchange of experiences and good practices, thus supporting the transition to an efficient and sustainable implementation of AI-CDSSs in medical practice.

From a practical perspective, this study provides clear recommendations for improving the implementation of AI-CDSSs in medical practice. A key recommendation is to optimize AI interfaces to reduce perceived technological complexity. The results suggest that professionals are more likely to accept AI-CDSSs when the systems are intuitive and their integration into clinical workflows does not disrupt existing activities. In this regard, developers should focus on creating solutions that minimize technological barriers and provide personalized support to users in the initial adoption phase. It is also recommended to actively involve end users in the development process to ensure that system functionalities are aligned with real needs in practice. Clear organizational policies and dedicated training sessions are also needed to facilitate the transition and encourage trust in these technologies.

The development of explainable and adaptable AI models, alongside dedicated professional training programs, represents a strategy for enhancing the acceptance and integration of these technologies in clinical practice. At the same time, this study highlights the need for ethical and methodological frameworks that appropriately reflect the use of AI in modern medicine, opening new paths for research on the long-term influence of AI-CDSSs on the quality of clinical decision-making and the interactions between professionals and technology.

This study presents certain limitations that must be considered when interpreting the results. Firstly, the sample of healthcare professionals is drawn exclusively from Romania, which may affect the generalizability of the conclusions. Factors such as digital infrastructure, AI regulation, and professional culture vary significantly across healthcare systems, potentially limiting the applicability of the findings at an international level. Otherwise, the sample selection is justified by the standard approach used in technology acceptance research models, such as PDA-UTAUT, which focus on user perceptions within a specific context. Another possible limitation of the study is that the recruitment method used could favor the inclusion of only professionals already familiar with artificial intelligence in the medical field, which may influence the perceptions expressed and reduce the degree of generalizability of the results.

Regarding future research directions, our results suggest several areas requiring further investigation. Firstly, analyzing the impact of cultural factors on AI adoption across different healthcare systems would be valuable, as technology acceptance may vary significantly based on normative and ethical differences between countries. Additionally, a promising research avenue would be to explore the effect of AI explainability on user trust, investigating which types of explanations are most effective in helping professionals understand and accept algorithmic decisions. Future studies could also examine the long-term impact of AI-CDSSs on clinical decision quality and patient safety, using longitudinal methodologies to assess the real-world effects of AI implementation in healthcare.

## 6. Conclusions

The integration of AI-CDSSs into the medical field represents a significant step in transforming the decision-making process. Nevertheless, their adoption is not an automatic process but, rather, one that depends on a series of interconnected factors. This study demonstrates that, beyond algorithmic performance, the perceptions of healthcare professionals, institutional support, the clarity of decision-making mechanisms, and technological accessibility directly influence the effective use of these systems.

One of the most relevant aspects identified is the importance of algorithmic transparency in fostering user trust in AI-CDSSs. A lack of clarity regarding how these systems generate clinical recommendations can lead to uncertainty and hesitancy in their use, suggesting that the developers of such technologies must prioritize explainability mechanisms. An AI system that provides detailed justifications and allows users to understand and adjust recommendations can facilitate acceptance in practice, reducing concerns regarding loss of control over medical decisions.

This study demonstrated that, while the perceived benefits of AI in medicine are widely acknowledged, they do not automatically translate into widespread adoption. Acceptance depends on additional factors such as familiarity with AI, the availability of empirical evidence regarding system effectiveness, and previous user experiences. This observation underscores the necessity of implementation strategies that include pilot programs, demonstration sessions, and a gradual adaptation period, allowing healthcare professionals to assess the tangible impacts of AI on their practice.

The integration of AI-CDSSs in the medical field cannot be reduced to the simple adoption of a new technology but involves a complex reconfiguration of the clinical decision-making context. The acceptance of these solutions largely relies on the organizational setup where they are implemented, which includes having clear rules, strong support from management, and a culture that encourages new ideas and teamwork across different fields. In the absence of these structuring elements, the level of skepticism among professionals remains high, even in the face of advanced technologies.

At the same time, the perception of operational accessibility plays a crucial role in the readiness for adoption: if these systems are considered cumbersome or difficult to learn, the probability of their integration into clinical routine decreases significantly. Therefore, intuitive design, smooth integration into existing flows, and continuous formative support become indispensable conditions for effective implementation.

Therefore, the digital transformation of medical practice through AI-CDSSs can only take place within a coherent strategy that articulates technological performance with professional training, institutional support, and clear governance. Only in such an integrated ecosystem can these systems reach their true potential—to strengthen the quality of clinical decision-making and patient safety.

## Figures and Tables

**Table 1:** Measurement items and descriptive statistics for all variables.

Constructs	Items
Perceived Benefits of AI-CDSSs (PBAI) [36,65,67,68]	(PBAI1) AI-CDSSs enhance diagnostic precision and mitigate the risk of medical errors.
	(PBAI2) AI-CDSSs optimize the clinical decision-making process, facilitating more rapid and evidence-informed medical decisions.
	(PBAI3) AI-CDSSs support the selection of optimal therapeutic interventions by leveraging advanced analytics and clinical evidence.
Technology Effort Cost (TEC) [13,26,43,66]	(TEC1) The implementation of AI-CDSSs necessitates substantial financial investments in infrastructure and maintenance.
	(TEC2) The effective use of AI-CDSSs requires extensive resource allocation for medical personnel training.
	(TEC3) The integration of AI-CDSSs into electronic health record systems entails significant costs.
	(TEC4) AI-CDSSs demand continuous technical support, leading to sustained operational expenditures.
Attitude Toward AI-CDSSs (ATAI) [21,60,65,66]	(ATAI1) AI-CDSSs represent a transformative innovation with the potential to enhance clinical practice.
	(ATAI2) I consider AI-CDSSs valuable tools for improving the quality of healthcare delivery.
	(ATAI3) I am confident in utilizing AI-CDSSs as an adjunct in clinical decision-making.
	(ATAI4) I am open to incorporating AI-CDSSs into my medical workflow.
Social and Institutional Influence (INF) [2,45,69,70]	(INF1) The adoption of AI-CDSSs is endorsed by my hospital/clinic administration, facilitating their implementation.
	(INF2) AI-CDSS is recommended by international clinical practice guidelines, reinforcing its credibility and utility.
Perceived Control and Transparency over AI (CTRL) [33,69,71,72,73]	(CTRL1) I retain the ability to modify AI-generated recommendations based on clinical judgment.
	(CTRL2) AI-CDSSs provide transparent explanations for their recommendations, enhancing my confidence in their reliability.
	(CTRL3) AI-CDSSs must maintain full transparency in their decision-making process to ensure safe and ethical integration into clinical practice.
Intention to Adopt AI-CDSSs (INT)	(INT1) I am actively interested in incorporating AI-CDSSs into my clinical practice and assessing their potential benefits.
	(INT2) I intend to initiate the implementation of AI-CDSSs, evaluating their alignment with my current medical practice.
	(INT3) Once integrated, I will routinely utilize an AI-CDSS as a decision-support tool in clinical practice.

**Table 2:** Sample characteristics.

Specialty	Number of Respondents	Percentage (%)	Justification
Radiology	95	21.59	AI-CDSSs are used in medical imaging interpretation, supporting rapid and precise diagnosis.
Oncology	75	17.04	AI-CDSSs assist in personalized treatments and biomarker analysis for early cancer detection.
Cardiology	70	15.91	AI optimizes ECG analysis, echocardiography, and cardiovascular risk prediction, supporting clinical decisions.
Emergency Medicine	65	14.77	AI-CDSSs support patient triage, medical image analysis, and rapid clinical decision-making.
Neurology	35	7.95	AI is used to diagnose stroke, epilepsy, and Parkinson’s disease, supporting clinical decisions.
Endocrinology	25	5.68	AI is applied in diabetology for automated monitoring and thyroid disease diagnosis algorithms.
Gastroenterology	25	5.68	AI is used in diagnostic decisions to support the detection of polyps and abnormalities in colonoscopy.
Pulmonology	20	4.54	AI-CDSS aids in CT analysis for lung diseases, diagnosing conditions such as Chronic obstructive pulmonary disease (COPD) and pulmonary fibrosis.
Robotic and Minimally Invasive Surgery	20	4.54	AI is used for intraoperative guidance and robotic assistance but less in direct clinical decision support.
Anesthesiology and Intensive Care	15	2.30	AI is used for critical patient monitoring, but its decision support role remains limited.
Total	440	100	

**Table 3:** Summary of measurement scales.

Constructs	Items	Mean	Standard Deviation	VIF	Factor Loading	Cronbach’s α	CR	AVE
**PBAI**	PBAI 1	4.21	0.90	1.62	0.78	0.79	0.86	0.62
	PBAI 2	4.18	0.88	1.68	0.81			
	PBAI 3	4.10	0.91	1.63	0.82			
**TEC**	TEC 1	3.72	0.95	1.41	0.76	0.71	0.83	0.64
	TEC 2	3.65	0.92	1.48	0.82			
	TEC 3	3.58	0.89	1.31	0.78			
	TEC 4	4.05	0.89	1.51	0.77			
**ATAI**	ATAI 1	4.08	0.85	1.63	0.79	0.81	0.87	0.72
	ATAI 2	4.15	0.81	1.82	0.85			
	ATAI 3	4.11	0.82	1.59	0.78			
	ATAI 4	4.07	0.84	1.65	0.82			
**INF**	INF 1	4.05	0.87	1.49	0.89	0.73	0.88	0.74
	INF 2	3.98	0.94	1.52	0.87			
**CTRL**	CTRL 1	4.12	0.91	2.18	0.84	0.88	0.92	0.76
	CTRL 2	4.08	0.87	2.75	0.91			
	CTRL 3	4.02	0.80	2.63	0.90			
**INT**	INT 1	4.12	0.83	1.33	0.79	0.744	0.85	0.67
	INT 2	3.95	0.79	1.76	0.85			
	INT 3	3.82	0.88	1.62	0.81			

**Table 4:** Fornell–Larcker criteria of the reflective constructs.

	ATAI	INT	TEC	CTRL	PBAI	INF
**ATAI**	0.805					
**INT**	0.673	0.812				
**TEC**	0.592	0.635	0.789			
**CTRL**	−0.321	−0.430	−0.231	0.905		
**PBAI**	0.538	0.417	0.455	−0.204	0.801	
**INF**	0.497	0.530	0.428	−0.175	0.414	0.881

**Table 5:** Cross-loadings of the reflective constructs.

	PBAI	TEC	ATAI	INF	CTRL	INT
**PBAI 1**	0.895	0.512	0.548	0.501	0.536	0.598
**PBAI 2**	0.872	0.498	0.524	0.487	0.521	0.573
**PBAI 3**	0.878	0.505	0.531	0.494	0.528	0.580
**TEC 1**	0.431	0.905	−0.327	−0.298	−0.317	−0.374
**TEC 2**	0.440	0.897	−0.312	−0.285	−0.299	−0.360
**TEC 3**	0.436	0.902	−0.319	−0.291	−0.305	−0.368
**TEC 4**	0.442	0.899	−0.321	−0.289	−0.307	−0.370
**ATAI 1**	0.512	−0.344	0.853	0.446	0.474	0.579
**ATAI 2**	0.519	−0.337	0.847	0.452	0.482	0.586
**ATAI 3**	0.526	−0.345	0.858	0.460	0.490	0.594
**ATAI 4**	0.534	−0.352	0.861	0.468	0.498	0.601
**INF 1**	0.473	−0.303	0.418	0.879	0.458	0.552
**INF 2**	0.480	−0.296	0.425	0.872	0.464	0.560
**CTRL 1**	0.451	−0.315	0.464	0.439	0.845	0.538
**CTRL 2**	0.459	−0.309	0.471	0.447	0.852	0.546
**CTRL 3**	0.466	−0.321	0.478	0.454	0.860	0.553
**INT 1**	0.521	−0.378	0.534	0.510	0.546	0.901
**INT 2**	0.526	−0.369	0.5442	0.518	0.554	0.908
**INT 3**	0.537	−0.382	0.550	0.526	0.562	0.915

**Table 6:** Heterotrait–Monotrait ratios of the reflective constructs.

	PBAI	TEC	ATAI	INF	CTRL	INT
**PBAI**	-					
**TEC**	0.49	-				
**ATAI**	0.51	0.47	-			
**INF**	0.55	0.50	0.54	-		
**CTRL**	0.53	0.46	0.49	0.56	-	
**INT**	0.58	0.51	0.55	0.59	0.57	-

## Data Availability

The raw data supporting the conclusions of this article will be made available by the authors on request.

## Abbreviations

The following abbreviations are used in this manuscript:

AI	Artificial intelligence
AI-CDSS	Artificial Intelligence Clinical Decision Support Systems
PLS-SEM	Partial Least Squares Structural Equation Modeling
TAM	Technology Acceptance Model
UTAUT	Unified Theory of Acceptance and Use of Technology
IS Success Model	Information Systems Success Model
PDA-UTAUT	Path Dependence-Augmented–Unified Theory of Acceptance and Use of Technology
PBAI	Perceived benefits of AI-CDSSs
TEC	Technology effort cost
ATAI	Attitude toward AI-CDSSs
INF	Social and institutional influence
CTRL	Perceived control and transparency over AI
INT	Intention to adopt AI-CDSSs
COPD	Chronic obstructive pulmonary disease
